# Supplementary material for: Hybrid Models and Biological Model Reduction with PyDSTool
Source: PLoS Comput Biol. 2012 Aug 9;8(8):e1002628. doi: 10.1371/journal.pcbi.1002628 (PMC3415397; doi:10.1371/journal.pcbi.1002628)
Supplement: Text S4 — Complete source code for the PyDSTool package (version 0.88.120504). Includes API documentation and help files linking to web pages. This file is identical to the current public release on Sourceforge.net. (ZIP) [file pcbi.1002628.s004.zip › PyDSTool/html/PyDSTool.Generator.Vode_ODEsystem'.Vode_ODEsystem-class.html]

xml version="1.0" encoding="ascii"?


PyDSTool.Generator.Vode\_ODEsystem'.Vode\_ODEsystem


| Home | Trees | Indices | Help | PyDSTool |
| --- | --- | --- | --- | --- |

Package PyDSTool ::
Package Generator ::
Module Vode\_ODEsystem' ::
Class Vode\_ODEsystem

[hide private]

[frames] | no frames]

# Class Vode\_ODEsystem

source code

```
           object --+            
                    |            
baseclasses.Generator --+        
                        |        
       baseclasses.ctsGen --+    
                            |    
         ODEsystem'.ODEsystem --+
                                |
                               Vode_ODEsystem
```

---

Wrapper for VODE, from SciPy.

Uses Python target language only for functional specifications.


|  |  |
| --- | --- |
| Instance Methods | [hide private] |

|  |  |
| --- | --- |
| AuxVars(self, t, xdict, pdict=None, asarray=True)  asarray is an unused, dummy argument for compatibility with Model.AuxVars | source code |

|  |  |
| --- | --- |
| Jacobian(self, t, xdict, pdict=None, asarray=True)  asarray is an unused, dummy argument for compatibility with Model.Jacobian | source code |

|  |  |
| --- | --- |
| JacobianP(self, t, xdict, pdict=None, asarray=True)  asarray is an unused, dummy argument for compatibility with Model.JacobianP | source code |

|  |  |
| --- | --- |
| Rhs(self, t, xdict, pdict=None, asarray=True)  asarray is an unused, dummy argument for compatibility with Model.Rhs | source code |

|  |  |
| --- | --- |
| \_\_del\_\_(self) | source code |

|  |  |
| --- | --- |
| \_\_init\_\_(self, kw)  x.\_\_init\_\_(...) initializes x; see x.\_\_class\_\_.\_\_doc\_\_ for signature | source code |

|  |  |
| --- | --- |
| \_debug\_snapshot(self, solver, dt, inputlist) | source code |

|  |  |
| --- | --- |
| addMethods(self)  Add Python-specific functions to this object's methods, accelerating them with psyco, if it is available. | source code |

|  |  |
| --- | --- |
| compute(self, trajname, dirn=`'``f``'`, ics=None)  This is an abstract class. | source code |

**Inherited from `ODEsystem'.ODEsystem`**:
`__getstate__`,
`__setstate__`,
`checkInitialConditions`,
`cleanupMemory`,
`haveJacobian`,
`haveJacobian_pars`,
`haveMass`,
`prepDirection`,
`set`,
`validateICs`

**Inherited from `baseclasses.ctsGen`**:
`validateSpec`

**Inherited from `baseclasses.Generator`**:
`__copy__`,
`__deepcopy__`,
`__repr__`,
`__str__`,
`addEvtPars`,
`checkArgs`,
`contains`,
`get`,
`getEventTimes`,
`getEvents`,
`info`,
`query`,
`resetEventTimes`,
`resetEvents`,
`setEventICs`,
`showAuxFnSpec`,
`showAuxSpec`,
`showEventSpec`,
`showSpec`

**Inherited from `baseclasses.Generator`** (private):
`_addEvents`,
`_auxfn_getindex`,
`_auxfn_globalindepvar`,
`_auxfn_heav`,
`_auxfn_if`,
`_auxfn_initcond`,
`_generate_ixmaps`,
`_infostr`,
`_kw_process_algparams`,
`_kw_process_allvars`,
`_kw_process_dispatch`,
`_kw_process_events`,
`_kw_process_fnspecs`,
`_kw_process_ics`,
`_kw_process_ignorespecial`,
`_kw_process_inputs`,
`_kw_process_pars`,
`_kw_process_pdomain`,
`_kw_process_reuseterms`,
`_kw_process_system`,
`_kw_process_target`,
`_kw_process_tdata`,
`_kw_process_tdomain`,
`_kw_process_tstep`,
`_kw_process_ttype`,
`_kw_process_varspecs`,
`_kw_process_vfcodeinserts`,
`_kw_process_xdomain`,
`_kw_process_xtype`,
`_makeBoundsEvents`,
`_register`,
`_set_for_hybrid_DS`

**Inherited from `object`**:
`__delattr__`,
`__getattribute__`,
`__hash__`,
`__new__`,
`__reduce__`,
`__reduce_ex__`,
`__setattr__`


|  |  |
| --- | --- |
| Class Variables | [hide private] |

**Inherited from `ODEsystem'.ODEsystem`** (private):
`_needKeys`,
`_optionalKeys`,
`_validKeys`

**Inherited from `baseclasses.Generator`** (private):
`_querykeys`


|  |  |
| --- | --- |
| Properties | [hide private] |

**Inherited from `object`**:
`__class__`


|  |  |
| --- | --- |
| Method Details | [hide private] |

|  |  |
| --- | --- |
| AuxVars(self, t, xdict, pdict=None, asarray=True) | source code |

asarray is an unused, dummy argument for compatibility with
Model.AuxVars

Overrides: ODEsystem'.ODEsystem.AuxVars

|  |  |
| --- | --- |
| Jacobian(self, t, xdict, pdict=None, asarray=True) | source code |

asarray is an unused, dummy argument for compatibility with
Model.Jacobian

Overrides: ODEsystem'.ODEsystem.Jacobian

|  |  |
| --- | --- |
| JacobianP(self, t, xdict, pdict=None, asarray=True) | source code |

asarray is an unused, dummy argument for compatibility with
Model.JacobianP

Overrides: ODEsystem'.ODEsystem.JacobianP

|  |  |
| --- | --- |
| Rhs(self, t, xdict, pdict=None, asarray=True) | source code |

asarray is an unused, dummy argument for compatibility with
Model.Rhs

Overrides: ODEsystem'.ODEsystem.Rhs

|  |  |
| --- | --- |
| \_\_del\_\_(self)  *(Destructor)* | source code |

Overrides: baseclasses.Generator.\_\_del\_\_

|  |  |
| --- | --- |
| \_\_init\_\_(self, kw)  *(Constructor)* | source code |

x.\_\_init\_\_(...) initializes x; see x.\_\_class\_\_.\_\_doc\_\_ for
signature

Overrides: object.\_\_init\_\_: *(inherited documentation)*

|  |  |
| --- | --- |
| addMethods(self) | source code |

Add Python-specific functions to this object's methods, accelerating
them with psyco, if it is available.

Overrides: ODEsystem'.ODEsystem.addMethods: *(inherited documentation)*

|  |  |
| --- | --- |
| compute(self, trajname, dirn=`'``f``'`, ics=None) | source code |

This is an abstract class.

Overrides: ODEsystem'.ODEsystem.compute: *(inherited documentation)*

  


| Home | Trees | Indices | Help | PyDSTool |
| --- | --- | --- | --- | --- |

|  |  |
| --- | --- |
| Generated by Epydoc 3.0.1 on Fri May 4 15:24:06 2012 | http://epydoc.sourceforge.net |
